# Obesity is associated with impaired postprandial pancreatic polypeptide secretion

**DOI:** 10.3389/fendo.2023.1192311

**Published:** 2023-06-02

**Authors:** Yanyun Zhao, Yue Zhou, Jingwei Chi, Kui Che, Yangang Wang, Wei Wang

**Affiliations:** ^1^ Department of Endocrinology and Metabolism, Affiliated Hospital of Qingdao University, Qingdao, China; ^2^ Medical Research Center, Qingdao Key Laboratory of Thyroid Diseases, Qingdao, China; ^3^ Department of Hematology, Affiliated Hospital of Qingdao University, Qingdao, China

**Keywords:** obesity, T2DM, pancreatic polypeptide, glucagon, islet function

## Abstract

**Objective:**

This study aims to compare the levels of serum pancreatic polypeptide (PP), insulin (INS), C-peptide (C-P), and glucagon (GCG) before and after glucose stimulation in type 2 diabetes mellitus (T2DM) patients with different body mass indexes (BMI), analyze the relevant factors associated with PP secretion, and further investigate the role of PP in the development of obesity and diabetes.

**Methods:**

Data were collected from 83 patients from the hospital. The subjects were divided into normal-weight group, overweight group, and obese group according to their BMI. All subjects were tested with the standard bread meal test (SBMT). PP and relevant parameters were measured, and the area under the curve (AUC) was calculated after 120 min of SBMT. AUC_pp_ (AUC of PP) was used as the dependent variable, and the potential influencing factors were used as independent variables for multiple linear regression analysis.

**Results:**

The obese and overweight groups had significantly lower PP secretion than the normal-weight group (485.95 pg·h/ml, 95% CI 76.16–895.74, *p* = 0.021; 664.61 pg·h/ml, 95% CI 285.46–1043.77, *p* = 0.001) at 60 min postprandial. PP secretion in the obese and overweight groups was also significantly lower than that in the normal-weight group (520.07 pg·h/ml, 95% CI 186.58–853.56, *p* = 0.003; 467.62 pg·h/ml, 95% CI 159.06–776.18, *p* = 0.003) at 120 min postprandial. AUC_pp_ was negatively associated with BMI (r = -0.260, *p* = 0.017) and positively associated with AUC_GCG_ (r = 0.501, *p<* 0.001). Multiple linear regression analysis showed that there was a linear correlation between AUC_GCG_, BMI, and AUC_pp_ (*p<* 0.001, *p* = 0.008). The regression equation was calculated as follows: AUC_pp_ = 1772.255–39.65 × BMI + 0.957 × AUC_GCG_ (R^2 = ^54.1%, *p<* 0.001).

**Conclusion:**

Compared with normal-weight subjects, overweight and obese subjects had impaired PP secretion after glucose stimulation. In T2DM patients, PP secretion was mainly affected by BMI and GCG.

**Clinical trial registry:**

The Ethics Committee of the Affiliated Hospital of Qingdao University.

**Clinical trial registration:**

http://www.chictr.org.cn, identifier ChiCTR2100047486.

## Highlights

• Compared with normal-weight subjects, overweight and obese subjects had impaired PP secretion after glucose stimulation.

• In T2DM patients, PP secretion was mainly affected by BMI and GCG levels.

• PP might potentially exert some protective effects on the function of islet cells.

## 1 Introduction

Pancreatic polypeptide (PP) is a pancreatic hormone which is mainly secreted by the endocrine pancreatic islet PP cells (also known as F cells or γ cells) (1) and belongs to the neuropeptide Y (NPY) family polypeptide (2–4). It mediates cell responses through the inhibitory G protein-coupled receptors of the NPY family (5). PP has a high affinity for the hypothalamic Y4 receptor (also known as PPYR4) and mediates biological activity after binding with it (1). A number of studies have reported that PP plays an important role in increasing energy consumption and reducing body weight by regulating food intake, inhibiting gallbladder movement, and inhibiting gastric emptying (6–10). Therefore, PP may induce satiety by activating the PPYR4 receptor, suggesting its potential anti-obesity effects (11).

Previous studies have shown that the relationship between PP and obesity is complex (12–14). In the early stage, it was shown that the body weight and fat accumulation in transgenic mice overexpressing PP were reduced due to decreased food intake and these changes were reversed after neutralization with anti-PP-containing serum (15). Women with bulimia nervosa have been reported to have reduced PP secretion after meals (16). Intravenous or intraperitoneal injection of PP increases the metabolic rate in obese mice, improves insulin resistance *in vivo*, and reduces hyperglycemia and hyperlipidemia (17). Further studies showed that exogenous administration of PP could reduce leptin levels in mice and resistin mRNA expression in white adipose tissue (18). Patients with Prader–Willi syndrome are known to have a slow postprandial PP response (10). Additionally, it has been reported that PP infusion reduces the food intake in Prader–Willi patients (19). Therefore, long-acting analogs of PP or inhibiting PP degradation have been suggested as potential strategies to treat obesity (3, 20, 21). Koska et al. (22) found that, although postprandial PP secretion was negatively correlated with body weight change, fasting PP level was positively correlated with the body weight change, which suggested that fasting and postprandial PP levels might have opposite effects on the risk of weight gain. On the other hand, others have shown evidence that fasting PP concentration is significantly positively correlated with visceral/liver fat area but not with subcutaneous fat area (23, 24). Some scholars have further proposed that fasting PP level can be regarded as an independent predictive factor of liver fat (25). However, it is still unclear whether PP can directly cause lipolysis and whether PP has different effects on the subcutaneous and visceral adipose tissues.

Therefore, we conducted a cross-sectional survey of the T2DM population with different BMIs to study the change in the secretion and factors influencing PP secretion in different stages of obesity. With this, we aimed to further analyze PP’s role in the pathogenesis of obesity and identify potential strategies for treating obesity and diabetes in the future.

## 2 Methods

### 2.1 Study population

A total of 83 hospitalized patients with type 2 diabetes mellitus (T2DM), including 41 women (49%) and 42 men (51%), were selected in the Department of Endocrinology of the Affiliated Hospital of Qingdao University from January 2020 to June 2020. All subjects were diagnosed with diabetes according to the WHO diagnostic criteria in 1999. The diagnostic criteria of diabetes mellitus were as follows: venous blood glucose ≥11.1 mmol/l 2 h after oral glucose tolerance test (OGTT), or fasting venous blood glucose (FBG) ≥7.0 mmol/l, or random venous blood glucose ≥11.1 mmol/l accompanied by typical diabetes symptoms.

The exclusion criteria were as follows: complicated with acute diabetic complications (such as diabetes ketoacidosis, hyperglycemia, and hyperosmolality); recently on thyroid hormone, and consumption of glucocorticoid and other drugs that could significantly influence blood glucose levels; gestational diabetes, type 1 diabetes mellitus (T1DM), secondary diabetes, special type diabetes, and other non-T2DM; consumption of glucagon-like peptide 1 (GLP-1) agonist, secretagogues, insulin, or dipeptidyl peptidase-IV (DPP-4) inhibitor drugs was used within 1 week before the experiment; diagnosed with polycystic ovary syndrome, Cushing disease, hyperthyroidism, acromegaly, and other autoimmune and endocrine diseases; complicated with severe kidney or liver diseases, cardiovascular disease, etc.; severe trauma, stress, or severe infection recently; complicated with PP cell tumor, pancreatic malignancy, acute or chronic pancreatitis, gastrointestinal ulcer, liver cirrhosis, depression, gastric cancer, and other diseases affecting PP secretion.

The study protocol was designed under the Declaration of Helsinki and was approved by the ethics committee of the Affiliated Hospital of Qingdao University, and all participants had provided written informed consent. The study was registered on http://www.chictr.org.cn/under the number ChiCTR2100047486.

### 2.2 Data collection

The age, gender, current and past medical history, alcohol consumption history, smoking history, and drug use history of the included subjects were recorded. The clinical examination was carried out by trained staff according to the standard guidelines. Diastolic blood pressure (DBP, mmHg) and systolic blood pressure (SBP, mmHg) of the right upper limb were measured after 15 min of rest, and the average of the two measurements was taken. The subjects were asked to take off their hats, shoes, and coats by a designated person before their waist circumference (cm), height (cm), and weight (kg) were measured. The measurements were taken after fasting and emptying the subject’s urine in advance. The waist circumference was measured as the circumference diameter of the midpoint between the anterior superior iliac spine and the costal margin. Body mass index was calculated using the formula (BMI) (kg/m^2^) = weight (kg)/height (m)^2^.

According to the 2013 guideline for the prevention and treatment of T2DM in China, the subjects were divided into three groups based on their BMI measurement: normal-weight group (18 kg/m^2^ ≤ BMI< 24 kg/m^2^), overweight group (24 kg/m^2^ ≤ BMI< 28 kg/m^2^), and obese group (BMI ≥28 kg/m^2^).

During laboratory examination, the following indicators were measured: FBG, glycosylated hemoglobin (HbA1c), C-peptide (C-P), insulin (INS), glucagon (GCG), blood lipids [high-density lipoprotein-cholesterol (HDL-c), low-density lipoprotein-cholesterol (LDL-c), total cholesterol (TC), triglycerides (TG), free fatty acid (FFA)], renal function [urea nitrogen (BUN), and creatinine (Cr)], liver function [aspartate aminotransferase (AST), alanine aminotransferase (ALT), and gamma-glutamyl-transferase (GGT)], and serum uric acid (UA).

From January 2020 to June 2020, T2DM subjects hospitalized in the Department of Endocrinology at the Affiliated Hospital of Qingdao University were subjected to the standard bread meal test (SBMT). After the fasting blood was extracted, the subjects ate a pair of bread made of 100-g Fuqiang flour (containing around 75–78 g of carbohydrates, 7–10 g of protein, 1–2 g of fat, and a total calorie equivalent to 75 g of glucose). Patients started timing themselves as soon as they took their first bite and had to consume the bread within 10–15 min. Additional food consumption or hard physical labor was not allowed during the test. The blood samples were drawn from the elbow vein at 60 and 120 min after SBMT to measure blood glucose, C-P, and INS at each time point. 2 ml of elbow vein blood was placed in an EDTA anticoagulant tube containing the DPP-4 inhibitor, which was then centrifuged in batches at 3,000 rpm/min for 10 min.

The upper serum was used for measuring PP and GCG. After collection, these samples were immediately stored in the laboratory refrigerator at -80°C until further examination. It is necessary to avoid repeated freezing and thawing during storage. The blood glucose level in each sample was measured using the glucose oxidase measurement kit (Olympus Automatic Biochemical Analyzer). The percentage of HbA1c was detected by monoclonal antibody agglutination reaction (Siemens DCA Vantage glycohemoglobin analyzer). The blood lipids, including HDL-C, LDL-C, TC, TG, and FFA, were measured by an enzymatic method (Olympus AU2700, Japan). The PP and GCG levels in each sample were detected using an enzyme-linked immunosorbent assay (ELISA) kit purchased from Shanghai Enzyme-linked Biotechnology Co., Ltd. More details regarding the PP ELISA assay are provided below. The PP ELISA assay had the lowest detectable concentration of less than 10 pg/ml. The detection range of PP ELISA was 93.75 to 3,000 pg/ml. In addition, the intra-assay and inter-assay coefficients of variability were less than 10% and 15%, respectively. The correlation coefficient R value between the linear regression of samples and the expected concentration was above 0.95. The PP ELISA assay was used to detect the total PP concentration. The specificity of the PP ELISA assay ranged from 85% to 115%. The assay could detect to some extent other peptides with a similar sequence as PP.

The INS and C-P of each sample were measured by radioimmunoassay (XH6020γ radio-immunocounter, Xi’an). The homeostasis model (HOMA-β) was used to evaluate the secretion function of islet β cells, which was calculated as 20× fasting insulin/(fasting glucose -3.5). The homeostasis model assessment of insulin resistance (HOMA-IR) was used to evaluate insulin sensitivity, and the formula was calculated as fasting glucose × fasting insulin/22.5. The area under the curve (AUC) of SBMT at 120 min for PP, INS, GCG, BG, and C-P was calculated as follows:

AUC_pp_=0.5×PP_0_+PP_60 + _0.5×PP_120_, AUC_ins_=0.5×INS_0_+ INS_60 + _0.5×INS_120_,

AUC_GCG_=0.5×GCG_0_+ GCG_60 + _0.5×GCG_120_, AUC_BG_=0.5×BG_0_+ BG_60 + _0.5×BG_120_,

AUC_C-P=_0.5×C-P_0_+ C-P_60 + _0.5×C-P_120_. Note: PP_0_, PP_60_, PP_120_, INS_0_, INS_60_, INS_120_, GCG_0_, GCG_60_, GCG_120_, BG_0_, BG_60_, BG_120_, C-P_0_, C-P_60_, and C-P_120_ represent the PP, INS, GCG, blood glucose, and C-P concentrations corresponding to fasting, 60 and 120 min after SBMT, respectively.

### 2.3 Statistical analysis

SPSS software version 22.0 (SPSS IBM Corporation, Armonk, NY, USA) was used for statistical analyses. The quantitative data that conformed to normal distribution were represented as mean ± standard deviation, and the quantitative data that conformed to non-normal distribution were expressed as median (quartile interval). The qualitative variable was described by rate or composition ratio. For the comparison of the means between different BMI groups, if the data conformed to the normal distribution and homogeneity of variance, the analysis of variance (ANOVA) was used. The comparison of general clinical data and hormone secretion indexes was used for one-way ANOVA of the three groups. Two-factor repeated-measure ANOVA was used for the overall analysis of PP, C-P, INS, and GCG groups at each time point. Multivariate ANOVA was used for paired comparison (the LSD method was used for multiple comparisons). Pearson correlation analysis was used for normally distributed data, and Spearman correlation analysis was used for non-normally distributed data. Multivariate stepwise regression analysis was used to build a regression model for AUC_pp_. When *p<* 0.05, the difference between groups was considered to be statistically significant.

## 3 Results

**Figure 1:**
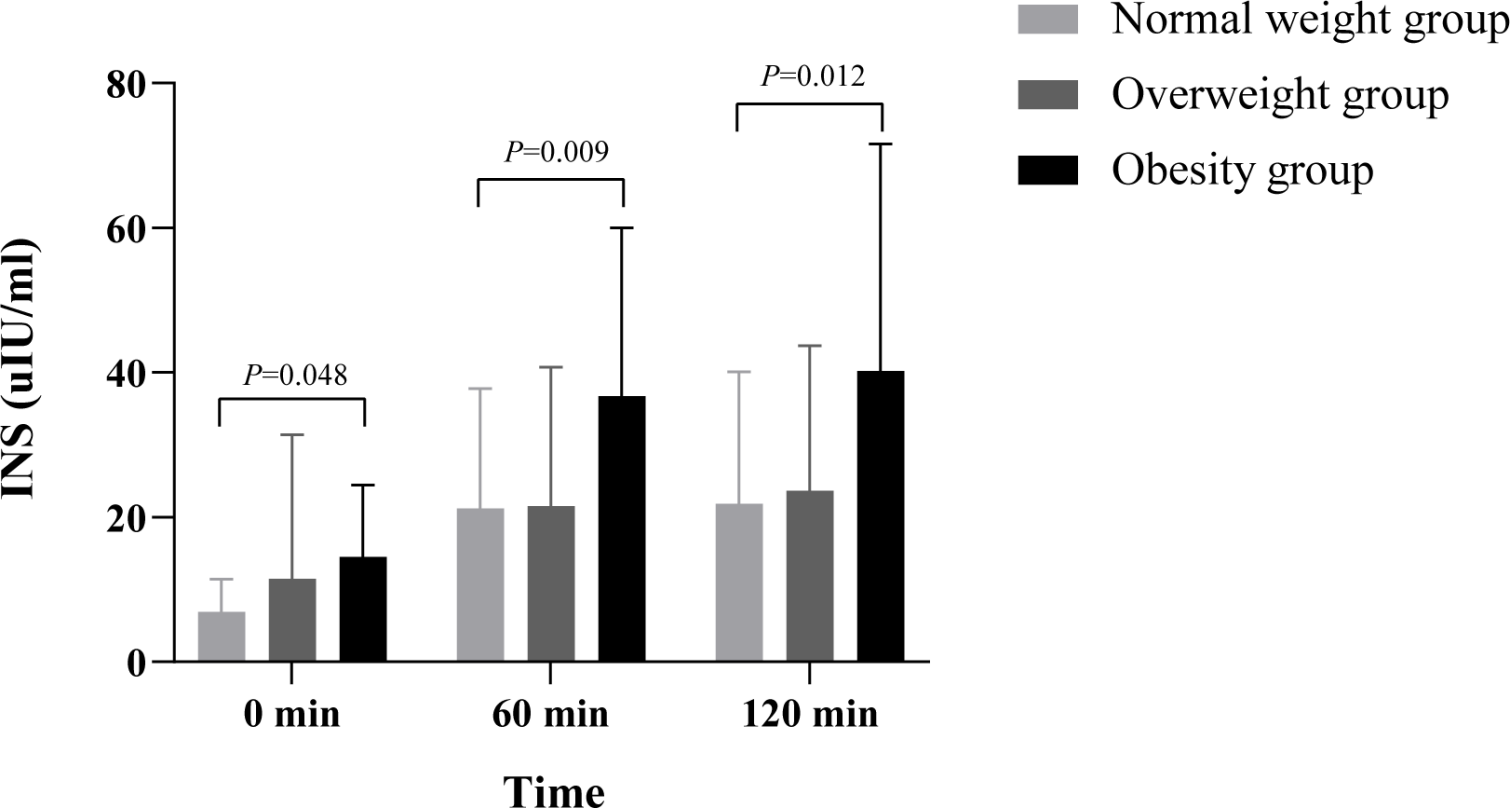
Comparison of INS between different BMI groups. The difference of INS between different time points and different BMI groups was statistically significant (p< 0.05), but the difference of time interaction between different BMI groups was not statistically significant (p > 0.05). Multiple comparisons among the groups showed that the fasting INS was in the obese group > overweight group > normal-weight group, and the difference between the obese group and normal-weight groups was statistically significant (7.60 µIU·h/ml, 95% confidence interval 0.06–15.15, p = 0.048). At postprandial 60 min, the INS in the obese group was significantly higher than that of the normal-weight group and the overweight group (15.54 µIU·h/ml, 95% confidence interval 4.55–26.53, p = 0.006; 15.17 µIU·h/ml, 95% confidence interval 4.35–25.99, p = 0.007). At postprandial 120 min, the INS in the obese group was significantly higher than that of the normal-weight group and the overweight group (16.55 µIU·h/ml, 95% confidence interval 3.53–29.57, p = 0.013; 18.36 µIU·h/ml, 95% confidence interval 5.54–31.19, p = 0.006). INS, insulin; BMI, body mass index.

**Figure 2:**
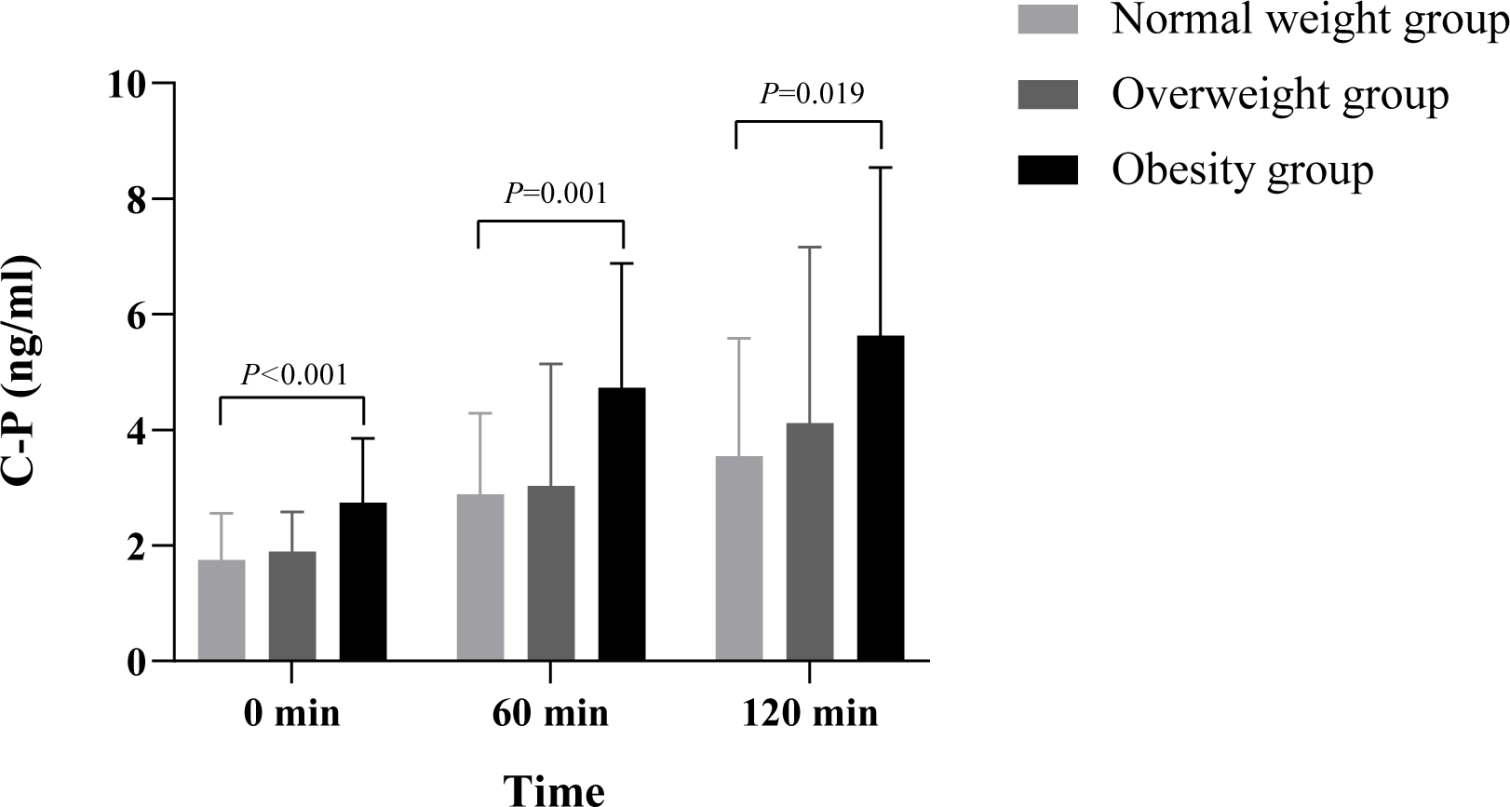
There were significant differences in GCG among the different time points of SBMT, different BMI groups, groups and time interactions (all p<0.05), suggesting that GCG secretion varies greatly in different obesity stages and different time points of SBMT, and the change trend over time is greatly affected by body weight. The GCG level of fasting and postprandial gradually increased with the increase of BMI. Multiple comparisons among groups showed that fasting GCG in the obese group was significantly higher than that normal-weight group and overweight group (126.56pg•h/mL, 95% confidence interval 79.85-173.27 p<0.001; 47.85pg•h/mL, 95% confidence interval 1.81-93.89, p=0.042). At 60 minutes after meal, the GCG in the obese group and the overweight group was significantly higher than that in the normal-weight group (124.41pg•h/mL, 95% confidence interval 56.17-192.64, p=0.001; 69.93pg•h/mL, 95% confidence interval 6.80-133.06, p=0.03), but there was no significant difference between obese group and overweight group (p>0.05). At 120 minutes after meal, there was significant difference in GCG level between obese group and normal-weight group (49.31pg•h/mL, 95% confidence interval 0.83-97.78, p=0.046), but there was no significant difference between obese group and overweight group, overweight group and normal-weight groups (p>0.05). GCG, glucagon; BMI, body mass index; SBMT, standard bread meal test.

**Figure 3:**
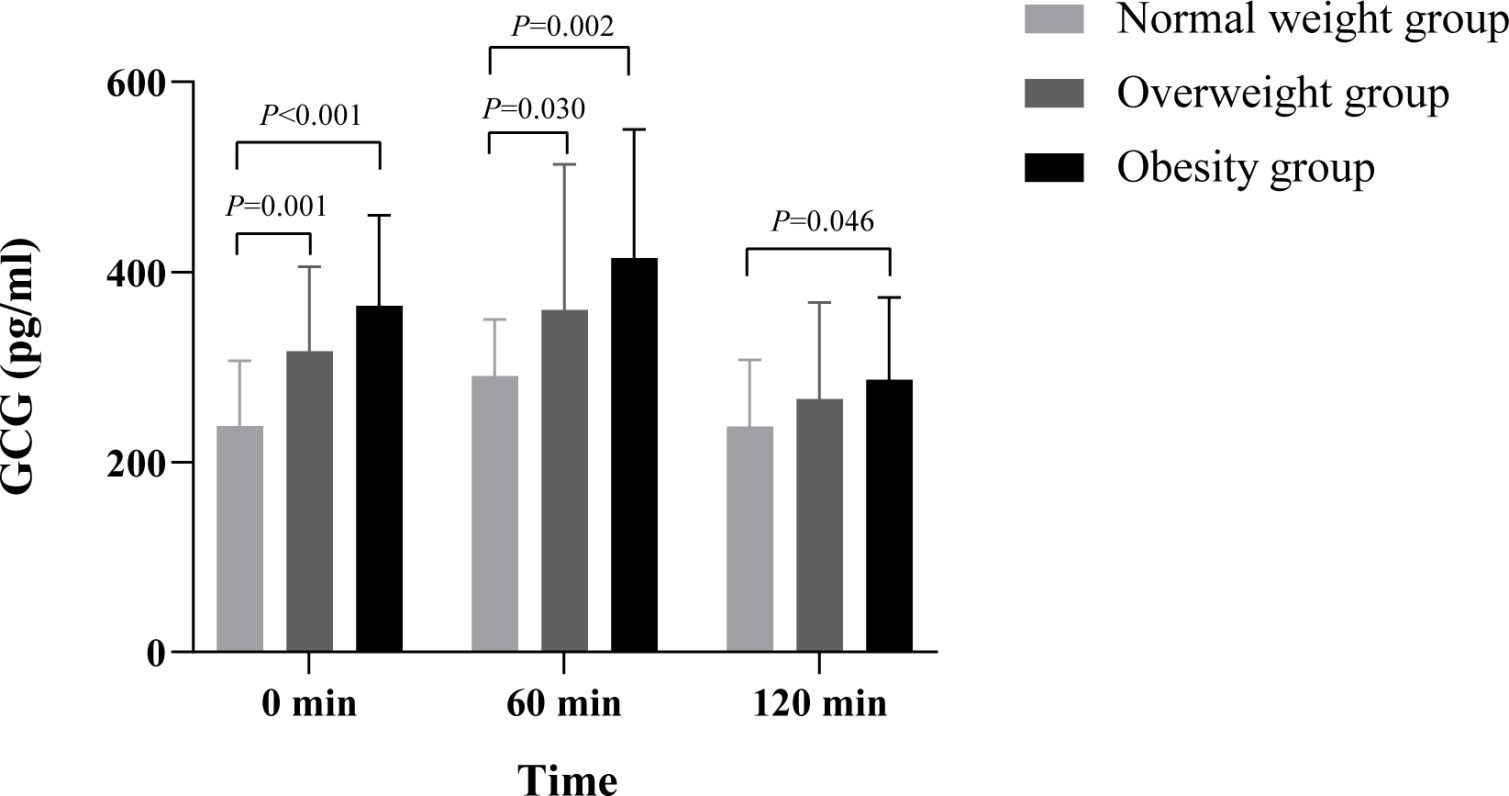
Comparison of C-P between different BMI groups. This study found that the difference of C-P between different time points and different BMI groups was statistically significant (p< 0.05), and there was no significant difference in the interaction between different BMI groups, groups, and time (p > 0.05). Multiple comparisons between groups showed that fasting C-P levels in the obese group were significantly higher than those in the normal-weight group and overweight group (0.99 ng·h/ml, 95% confidence interval 0.51–1.47, p< 0.001; 0.83 ng·h/ml, 95% confidence interval 0.36–1.31, p = 0.001). At 60 min after meal, the C-P levels in the obese group were significantly higher than those in the normal-weight group and overweight group (1.70 ng·h/ml, 95% confidence interval 0.65–2.74, p = 0.002; 1.84 ng·h/ml, 95% confidence interval 0.81–2.87, p = 0.001). At 120 min after meal, the C-P levels in the obese group were significantly higher than those in the normal-weight group and overweight group (1.51 ng·h/ml, 95% confidence interval 0.03–2.99, p = 0.046; 2.08 ng·h/ml, 95% confidence interval 0.62-3.54, p = 0.006). The difference between the normal weight group and overweight group was not significant (p > 0.05). C-P, C-peptide; BMI, body mass index.

**Table 1** shows the general clinical characteristics and biochemical indicators of T2DM patients in the normal-weight group, overweight group, and obese group, including the comparison of the trends for INS, GCG, and C-P among the different BMI groups (**Figures 1**–**3**). Moreover, the comparison of AUC_ins_, AUC_GCG_, and AUC_C-P_ among different BMI groups of T2DM patients is shown in **Supplementary Figures 1**-**3**.

**Table 1:** Comparison of clinical data with different BMI groups $(\bar{x}$ ± s) .

Clinical features	Normal-weight group (29)	Overweight group (31)	Obese group (23)	*p* value
Age (years)	58.83 ± 7.43	54.35 ± 10.57	55.00 ± 10.72	0.168
Gender (female%)	19(65.5%)	13(41.9%)	9(39.1%)	0.097
BMI (kg/m^2^)	21.73 ± 1.34^bc^	26.48 ± 0.93^ac^	29.86 ± 2.12^ab^	<0.001
Waist circumference (cm)	83.93 ± 7.67^bc^	96.91 ± 4.77^ac^	104.08 ± 6.03^ab^	<0.001
SBP (mmHg)	131.31 ± 19.22	139.03 ± 18.30	136.74 ± 20.13	0.287
DBP (mmHg)	73.93 ± 11.61^bc^	81.87 ± 10.02^a^	80.70 ± 10.29^a^	0.012
HbA1c (%)	7.93 ± 2.11	8.38 ± 1.82	8.07 ± 1.87	0.659
FBG (mmol/L)	6.50 ± 1.94	7.74 ± 4.90	7.59 ± 2.44	0.341
LDL-c (mmol/L)	2.85 ± 0.93	3.01 ± 0.85	2.47 ± 0.74	0.073
HDL-c (mmol/L)	1.23 ± 0.30	1.12 ± 0.22	1.10 ± 0.23	0.134
TC (mmol/L)	4.55 ± 1.25	4.81 ± 1.07	4.16 ± 0.99	0.109
TG (mmol/L)	1.58 ± 1.36	1.88 ± 1.08	1.81 ± 0.80	0.570
FFA (mmol/L)	0.33 ± 0.18	0.40 ± 0.18	0.39 ± 0.25	0.381
UA (mmol/L)	308.79 ± 69.83^c^	330.17 ± 110.36	363.78 ± 99.03^a^	0.126
Cr (µmol/L)	49.21 ± 17.14	58.98 ± 32.97	56.43 ± 16.70	0.282
BUN (mmol/L)	6.00 ± 1.56	6.32 ± 3.02	5.86 ± 1.85	0.753
ALT (U/L)	19.90 ± 9.17	29.0 ± 22.61	27.35 ± 21.81	0.278
AST (U/L)	16.24 ± 4.12^b^	20.68 ± 9.18^a^	18.17 ± 6.54	0.145
HOMA-IR	2.19 ± 2.39^c^	2.66 ± 1.70	5.01 ± 3.84^a^	0.001
HOMA-β	60.39 ± 42.25^c^	58.19 ± 41.14	97.89 ± 91.63^a^	0.207
PP_0_ (pg·h/mL)	1,754.90 ± 458.02	1,690.90 ± 704.30	1,648.96 ± 605.18	0.813
PP_60_ (pg·h/mL)	2,665.43 ± 577.62^bc^	2,000.82 ± 733.10^a^	2,179.48 ± 905.69^a^	0.003
PP_120_ (pg·h/mL)	2,009.81 ± 600.75^bc^	1,542.20 ± 594.62^a^	1,489.74 ± 606.96^a^	0.003
AUC_pp_ (pg·h/mL)	909.56 ± 137.49^bc^	723.47 ± 249.18^a^	749.77 ± 272.51^a^	0.004
GCG_0_ (pg·h/mL)	238.12 ± 68.33^bc^	316.83 ± 88.78^ac^	364.68 ± 94.90^ab^	<0.0001
GCG_60_ (pg·h/mL)	290.37 ± 59.73^bc^	360.30 ± 153.14^a^	414.78 ± 135.32^a^	0.002
GCG_120_ (pg·h/mL)	237.60 ± 69.72^c^	266.46 ± 101.39	286.91 ± 86.41^a^	0.128
AUC_GCG_ (pg·h/mL)	528.24 ± 73.70^bc^	651.95 ± 219.58^a^	740.58 ± 203.44^a^	<0.0001
INS_0_ (µIU·h/mL)	6.94 ± 4.50^c^	11.51 ± 19.86	14.54 ± 9.90^a^	0.132
INS_60_ (µIU·h/mL)	21.20 ± 16.59^c^	21.57 ± 19.18^c^	36.74 ± 23.25^ab^	0.009
INS_120_ (µIU·h/mL)	21.87 ± 18.22^a^	23.69 ± 20.04^c^	40.23 ± 31.38^ab^	0.012
AUC_ins_ (µIU·h/mL)	36.51 ± 27.00^c^	38.26 ± 36.12	64.13 ± 38.65^a^	0.001
C-P_0_ (ng·h/mL)	1.75 ± 0.81^c^	1.90 ± 0.68	2.74 ± 1.11^a^	< 0.001
C-P_60_ (ng·h/mL)	2.89 ± 1.40^c^	3.03 ± 2.11	4.73 ± 2.15^a^	0.001
C-P_120_ (ng·h/mL)	3.55 ± 2.03^c^	4.12 ± 3.04	5.63 ± 2.91^a^	0.019
AUC_cp_ (ng·h/mL)	5.62 ± 2.57^c^	5.97 ± 3.89	8.91 ± 3.66^a^	0.008

a p< 0.05, compared with the normal-weight group.

b p< 0.05, compared with the overweight group.

c p< 0.05, compared with the obesity group.

ALT, alanine transaminase; AST, aspartate transaminase; AUC_cp_, AUC_GCG_, AUC_ins_, AUC_pp_, area under the curve for C-peptide, glucagon, insulin, pancreatic polypeptide, respectively; BMI, body mass index; BUN, blood urea nitrogen; Cr, creatinine; DBP, diastolic blood pressure; FBG, fasting blood glucose; FFA, free fatty acid; HbA1C, glycosylated hemoglobin; HDL-c, high-density lipoprotein cholesterol; HOMA-β, homeostatic model assessment of beta-cell function; HOMA-IR, homeostatic model assessment of insulin resistance; LDL-c, low-density lipoprotein cholesterol; SBP, systolic blood pressure; TC, total cholesterol; TG, triglyceride; UA, serum uric acid.

**Figure 4:**
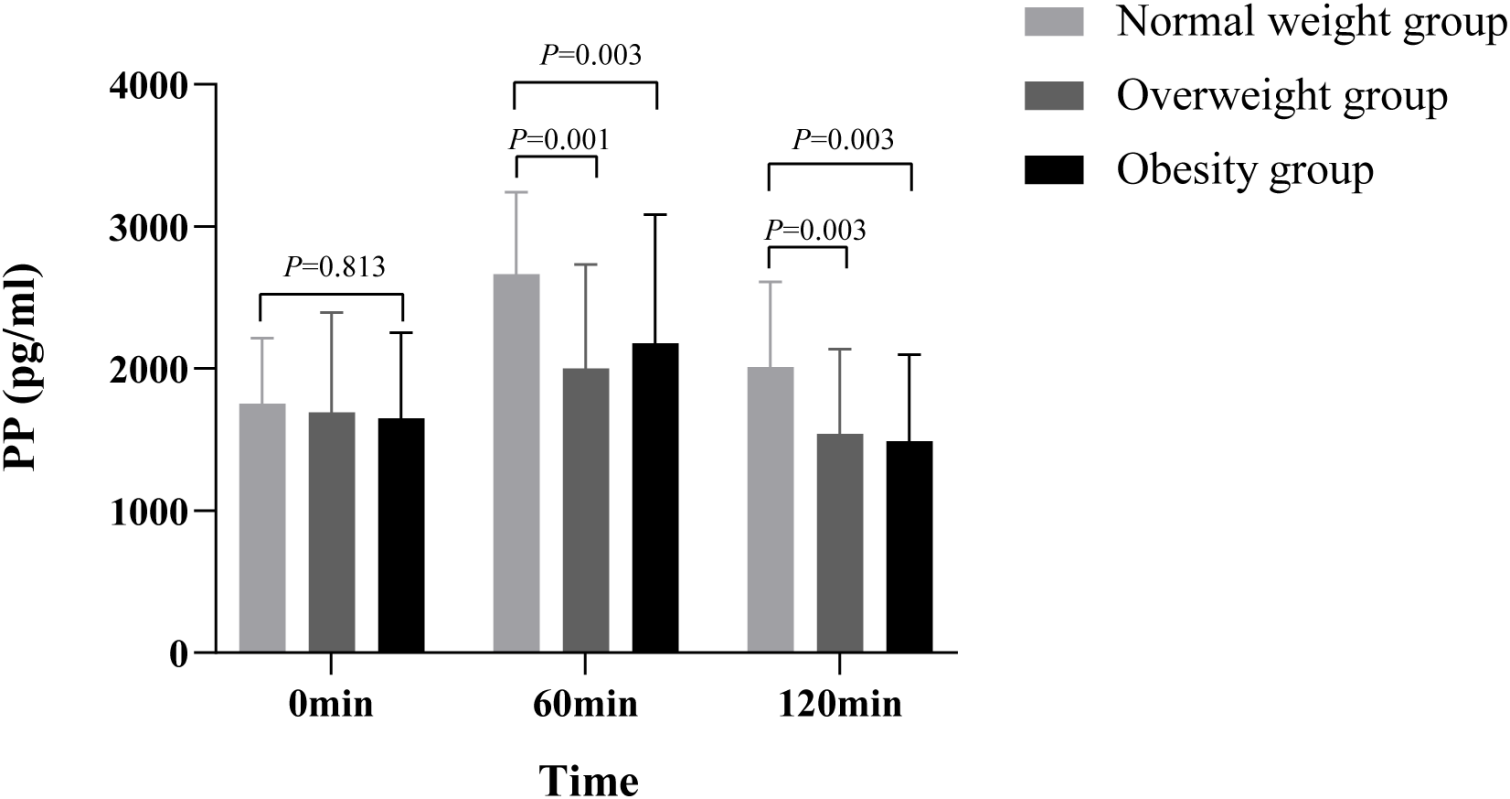
Comparison of PP between different BMI groups There were significant differences in PP among the different time points of SBMT, different BMI groups, groups, and time interactions (all p< 0.05), suggesting that PP secretion varies greatly in different obesity stages and different time points of SBMT, and the change trend over time is greatly affected by body weight. Multiple comparisons between groups showed that there was no significant difference in fasting PP between different BMI groups (p > 0.05). At postprandial 60 min, PP secretion of the obesity and the overweight group was significantly lower than that of the normal-weight group (485.95 pg·h/ml, 95% confidence interval 76.16–895.74, p = 0.021; 664.61 pg·h/ml, 95% confidence interval 285.46–1043.77, p = 0.001). At 120 min after SBMT, PP secretion of the obesity and the overweight group was also significantly lower than that of the normal-weight group (520.07 pg·h/ml, 95% confidence interval 186.58–853.56, p = 0.003; 467.62 pg·h/ml, 95% confidence interval 159.06–776.18, p = 0.003). PP, pancreatic polypeptide; BMI, body mass index; SBMT, standard bread meal test.

The comparison of PP trends among the different BMI groups of T2DM patients is presented in **Table 1** (**Figure 4**). There were significant differences in PP at the different time points after SBMT, different BMI groups, groups, and time interactions (all *p<* 0.05). These results suggested that PP secretion varied greatly in different stages of obesity and different time points after SBMT, and the change trend over time was greatly affected by the body weight. Multiple comparisons between groups showed that there was no significant difference in fasting levels of PP between the different BMI groups (*p* > 0.05). At 60 min after the meal, PP secretion in the obese and overweight groups was significantly lower than that of the normal-weight group (485.95 pg·h/ml, 95% CI 76.16–895.74, *p* = 0.021; 664.61 pg·h/ml, 95% CI 285.46–1043.77, *p* = 0.001). At 120 min after SBMT, PP secretion in the obese and overweight groups was also significantly lower than that of the normal-weight group (520.07 pg·h/ml, 95% CI 186.58–853.56, *p* = 0.003; 467.62 pg·h/ml, 95% CI 159.06–776.18, *p* = 0.003). AUC_pp_ in the obese and overweight groups was lower than that in the normal-weight group, and the differences were statistically significant (159.79 pg·h/ml, 95% CI 35.14–284.44, *p* = 0.013; 186.08 pg·h/ml, 95% CI 70.75–301.41, *p* = 0.002) (**Supplementary Figure 4**).

The analysis of the correlation between PP and AUC_pp_ at different time points is shown in **Table 2**. The results demonstrated that fasting PP was significantly positively correlated with AUC_GCG_ (r = 0.564, *p<* 0.001). At 60 min after the meal, PP level was significantly negatively correlated with BMI, HbA1c, LDL-c, and TC (r = -0.255, r = -0.249, r = -0.260, r = -0.234, all *p<* 0.05) and was significantly positively correlated with AUC_GCG_ and AUC_c-p_ (r = 0.421, r = 0.253, all *p<* 0.05). At 120 min after the meal, PP level was significantly negatively correlated with BMI, waist circumference, fasting blood glucose, and HOMA-IR (r = -0.271, r = -0.265, r = -0.235, r = -0.253, all *p<* 0.05) and was significantly positively correlated with AUC_GCG_ (r = 0.299, *p<* 0.05). AUC_PP_ was significantly negatively correlated with BMI (r = -0.260, *p<* 0.05) and was significantly positively correlated with AUC_GCG_ (r = 0.501, *p<* 0.05). The basal PP level and glucose-stimulated PP secretion level were not significantly correlated with the patient’s diabetes duration, age, HDL-c, TG, FFA, UA, HOMA-β, and AUC_ins_.

**Table 2:** Analysis of related factors of PP in type 2 diabetic patients.

Clinical features	PP_0_		PP_60_		PP_120_		AUC_pp_	
	r	*p*	r	*p*	r	*p*	r	*p*
Age (years)	-0.149	0.178	0.006	0.956	-0.136	0.222	-0.070	0.531
Diabetes course (years)	-0.008	0.941	-0.025	0.820	0.003	0.981	-0.018	0.871
BMI (kg/m^2^)	-0.074	0.507	-0.255	0.020^*^	-0.271	0.013^*^	-0.260	0.017^*^
Waist circumference (cm)	-0.069	0.600	-0.153	0.242	-0.265	0.041^*^	-0.186	0.155
SBP (mmHg)	-0.020	0.858	-0.134	0.228	-0.135	0.223	-0.130	0.242
DBP (mmHg)	0.124	0.263	-0.151	0.172	-0.055	0.624	-0.084	0.452
HbA1c (%)	-0.067	0.544	-0.249	0.023^*^	-0.067	0.547	-0.200	0.070
FBG (mmol/L)	-0.037	0.738	-0.186	0.091	-0.235	0.033^*^	-0.196	0.076
LDL-c (mmol/L)	-0.161	0.146	-0.260	0.017^*^	-0.010	0.931	-0.251	0.051
HDL-c (mmol/L)	-0.151	0.175	-0.184	0.098	-0.023	0.841	-0.165	0.138
TC (mmol/L)	-0.170	0.127	-0.234	0.035^*^	0.020	0.862	-0.191	0.085
TG (mmol/L)	0.051	0.647	-0.082	0.462	0.058	0.605	-0.026	0.817
FFA (mmol/L)	0.074	0.510	0.035	0.754	-0.011	0.919	0.039	0.730
UA (mmol/L)	-0.072	0.519	0.022	0.845	-0.112	0.315	-0.034	0.764
HOMA-IR	-0.111	0.328	-0.030	0.793	-0.253^*^	0.023	-0.061	0.594
HOMA-β	-0.058	0.610	0.197	0.081	-0.009	0.939	0.133	0.241
AUC_GCG_ (pg·h/mL)	0.564	<0.001^***^	0.421	<0.001^***^	0.299	0.006^**^	0.501	<0.001^***^
AUC_ins_ (µIU·h/mL)	-0.041	0.713	0.209	0.061	-0.026	0.818	0.148	0.186
AUC_cp_ (ng·h/mL)	0.021	0.848	0.253	0.021^*^	-0.041	0.714	0.179	0.106

^*^p< 0.05, ^**^p< 0.01, ^***^p< 0.001.

AUC_cp_, AUC_GCG_, AUC_ins_, area under the curve for C-peptide, glucagon, and insulin, respectively; BMI, body mass index; DBP, diastolic blood pressure; FBG, fasting blood glucose; FFA, free fatty acid; HbA1C, glycosylated hemoglobin; HDL-c, high-density lipoprotein cholesterol; HOMA-β, homeostatic model assessment of beta cell function; HOMA-IR, homeostatic model assessment of insulin resistance; LDL-c, low-density lipoprotein cholesterol; SBP, systolic blood pressure; TC, total cholesterol; TG, triglyceride; UA, uric acid.

We used AUC_PP_ as the dependent variable and the potentially possible elements such as BMI and AUC_GCG_ as the independent variables for stepwise fitting. After adjusting for age, TG, TC, ALT, AST, HDL-C, LDL-C, SBP, and DBP, the results from the multiple linear regression analysis demonstrated that there was a linear correlation between BMI, AUC_GCG_, and AUC_PP_ (all *p<* 0.001). The regression equation was calculated as follows: AUC_pp_ = 1772.255–39.65 × BMI + 0.957 × AUC_GCG_ (R^2 = ^54.1%, *p<* 0.001).

## 4 Discussion

PP belongs to the family of gut hormones secreted mainly by the endocrine PP cells (also known as F cells or γ cells) of the pancreas and the colon (1, 6) and is the endogenous ligand of hypothalamic neuropeptide Y4 receptors (NPY4R or Y4) (26). PP is released into the blood after meals, and its release is triggered by intestinal stimulation such as glucose (27), fat (28), and protein (29). PP secretion is mainly controlled by the vagus nerve (30). Sive et al. (31) also found that the sympathetic nervous system also played a role in regulating PP secretion. In addition, studies have shown that cholecystokinin (CCK) is a stimulant of PP secretion, whereas somatostatin (SST) is an inhibitor of PP secretion (32). Moreover, GIP has also been reported to stimulate PP secretion (33) but the exact molecular mechanism has not yet been identified. In addition, it has been reported that gastric fundus dilation and intraduodenal infusion of bile pancreatic juice can also stimulate PP secretion (34, 35).

In this study, we found that obesity did not affect basal PP secretion but was associated with decreased PP secretion after glucose stimulation in the Chinese T2DM population. The results were consistent with a range of previous findings including the one reported by Lagae et al. (36), which showed that there were relatively few PP-producing cells in the islets of genetically obese mice. Ueno et al. (15) subsequently identified that transgenic mice overexpressing PP lost body weight and fat accumulation due to decreased food intake, and the above changes were reversed after immune neutralization with anti-PP containing serum. Sainsbury et al. (17) also reported that intravenous or intraperitoneal injection of PP could increase the metabolic rate of obese mice, improve insulin resistance *in vivo*, and relieve hyperglycemia and hyperlipidemia. In this study, the analysis of factors affecting PP secretion also consistently revealed that PP secretion after glucose stimulation was negatively correlated with BMI in this study. Studies have shown that the signal from the vagus nerve was interrupted in animal models of obesity (37, 38). The activation of the vagus nerve has been shown to reduce fat accumulation by regulating gastrointestinal motility and gastrointestinal hormone secretion (39–42). The secretion of PP is vagal-cholinergic dependent (30, 43), so the decreased parasympathetic nerve function caused by obesity may lead to a reduction in the secretion of PP. Combined with the results of previous and current studies, we assume that the reduced postprandial secretion of PP is associated with the risk of weight gain.

It is noteworthy that although our results showed that there was no significant difference in the basal level of PP between the different BMI groups, there was evidence that the fasting PP level was not significantly correlated with the subcutaneous fat area (SFA) and that it was significantly positively correlated with the visceral/liver fat area (VFA) (23, 24). Some studies further proposed that fasting PP level could be regarded as an independent predictor of visceral or liver fat (25). However, it is still unclear whether PP has different effects on the subcutaneous versus the visceral adipose tissue. On the other hand, PP, as a gastrointestinal hormone, plays an important physiological role in increasing energy expenditure and weight loss by reducing appetite, inhibiting gastric emptying, and increasing satiety (6–10). PP has also been shown to reduce leptin levels *in vivo* and resistin mRNA expression in white adipose tissue (18). The above data indicate that PP plays an important role in body weight regulation. Therefore, the use of long-lasting PP analogs or inhibition of PP degradation has emerged as an effective strategy to treat obesity in recent years (3, 20, 21), which may be clinically relevant.

The analysis of factors related to PP secretion in this study showed that there was no significant correlation between INS and PP secretion at baseline but there was a significant positive correlation between them 60 min after SBMT. Consistent with this, Schwartz et al. (20) previously reported that excessive proliferation of PP cells occurred in the pancreatic tissue of insulinoma patients. Subsequently, Weyer et al. (44) reported that the Pima Indian population associated with a high risk of T2DM had obvious hyperinsulinemia and elevated PP levels. Some studies have demonstrated that β-cell dysfunction is associated with PP cell injury in chronic pancreatitis patients (45–47). Exogenous administration of PP can reverse hepatic insulin resistance and improve blood glucose control in patients with chronic pancreatitis (48) and pancreatectomy (49). A study (50) has confirmed that PP-induced Y4 receptor activation protects β cells free from apoptosis. In addition, PP has been reported to improve insulin sensitivity by improving the effectiveness of liver insulin receptors (34), which suppresses the downregulation of insulin receptors induced by hyperinsulinemia (51). In this study, the significantly negative correlation (*p* = 0.023) between HOMA-IR and PP concentrations at 120 postprandial minutes in T2DM patients was again consistent with the above point, also suggesting that the decreased level of PP secretion is related to the increased risk of insulin resistance. These observations indicate that PP has important beneficial effects on the survival and function of islet cells, suggesting the potential role of PP in the treatment of T2DM patients in the clinic.

In conclusion, compared with normal-weight people, overweight and obese people have impaired PP secretion after glucose stimulation. This reaction may be masked by increased PP secretion caused by abnormal glucose tolerance. In addition, reduced postprandial PP secretion is associated with GCG levels and high BMI in T2DM patients. PP may have potential beneficial effects on the survival and function of islet cells. It is important to note that this study was conducted in T2DM patients, so the conclusions might not apply to non-diabetic subjects. In addition, another limitation of this study is the failure to detect CCK, SST, and GIP to further analyze their effects on PP secretion. Moreover, this study, as a cross-sectional study, is not sufficient to confirm the causal relationship between PP and obesity. Thus, further studies exploring of specific mechanisms are needed in the future. However, our research reveals a novel role of PP in the pathogenesis of obesity and diabetes. Thus, targeting PP secretion may offer a potential therapeutic strategy for the treatment of obesity and diabetes in the clinic.

## Data availability statement

The raw data supporting the conclusions of this article will be made available by the authors, without undue reservation.

## Ethics statement

Written informed consent was obtained from the individual(s), and minor(s)’ legal guardian/next of kin, for the publication of any potentially identifiable images or data included in this article.

## Author contributions

YW, WW, and YZ did the study design. YYZ, YZ, WW, and YW contributed to the data collection. YYZ and YZ analyzed the study data. YYZ and YZ wrote the manuscript and created the figures. JC and KC reviewed and edited the manuscript. YYZ and YZ contributed to the figure and table editing. All authors reviewed the manuscript, approved the final draft, and agreed to submit it for publication.

## Supplementary material

Supplementary Figure 1 The area under curves for the INS. Multiple comparisons between groups showed that AUC_ins_ in the obese group was higher than that in the normal-weight group, and the differences were statistically significant (2.95uIU·h/mL, 95% confidence interval 1.07-4.83, p=0.003). And AUC_ins_ in the obese group was higher than that in the overweight group, and the differences were statistically significant (3.30uIU·h/mL, 95% confidence interval 1.45-5.15, p=0.001). The difference between overweight group and normal group was not significant (P>0.05). AUC_ins_, the area under the curve of insulin

Supplementary Figure 2 The area under curves for the GCG. Multiple comparisons between groups showed that AUC_GCG_ in the obese and overweight group was higher than that in the normal-weight group, and the differences were statistically significant (212.34pg·h/mL, 95% confidence interval 113.94-310.74, p<0.001; 123.72pg·h/mL, 95% confidence interval 32.67-214.76, p=0.008). The difference between obese group and overweight group was not significant (P>0.05). AUC_GCG_, the area under the curve of glucagon

Supplementary Figure 3 The area under curves for the C-P. Multiple comparisons between groups showed that AUC_C-P_ in the obese group was higher than that in the normal-weight group, and the differences were statistically significant (27.62ng·h/mL, 95% confidence interval 8.55-46.69, p=0.005). AUC_C-P_ in the obese group was higher than that in the overweight group, and the differences were statistically significant (25.87ng·h/mL, 95% confidence interval 7.08-44.65, p=0.008). The difference between overweight group and normal-weight group was not significant (P>0.05). AUC_C-P_, the area under the curve of C-peptide

Supplementary Figure 4 The area under curves for the PP. Multiple comparisons between groups showed that AUC_pp_ in the obese and overweight group was lower than that in the normal-weight group, and the differences were statistically significant (159.79pg·h/mL, 95% confidence interval 35.14-284.44, p=0.013; 186.08pg·h/mL, 95% confidence interval 70.75-301.41, p=0.002). The difference between obese group and overweight group was not significant (P>0.05). AUC_pp_, the area under the curve of pancreatic polypeptide
