# Supplementary figures and images for: Obesity is associated with impaired postprandial pancreatic polypeptide secretion
Source: Front Endocrinol (Lausanne). 2023 Jun 2;14:1192311. doi: 10.3389/fendo.2023.1192311 (PMC10273268; doi:10.3389/fendo.2023.1192311)

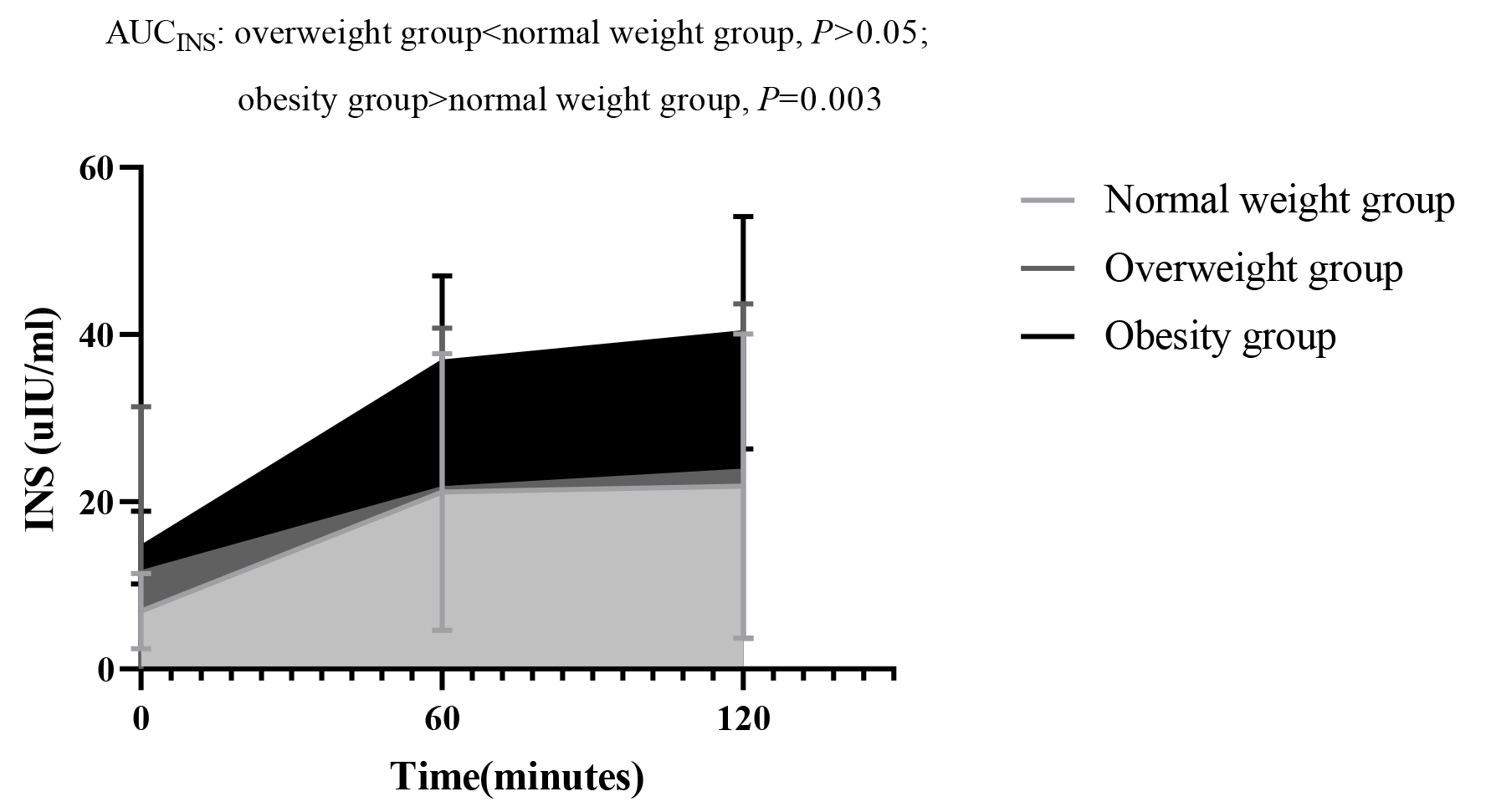

Supplement: Supplementary Figure 1 — The area under curves for the INS. Multiple comparisons between groups showed that AUCins in the obese group was higher than that in the normal-weight group, and the differences were statistically significant (2.95uIU·h/mL, 95% confidence interval 1.07-4.83, p=0.003). And AUCins in the obese group was higher than that in the overweight group, and the differences were statistically significant (3.30uIU·h/mL, 95% confidence interval 1.45-5.15, p=0.001). The difference between overweight group and normal group was not significant (P>0.05). AUCins, the area under the curve of insulin [file Image_1.tif]

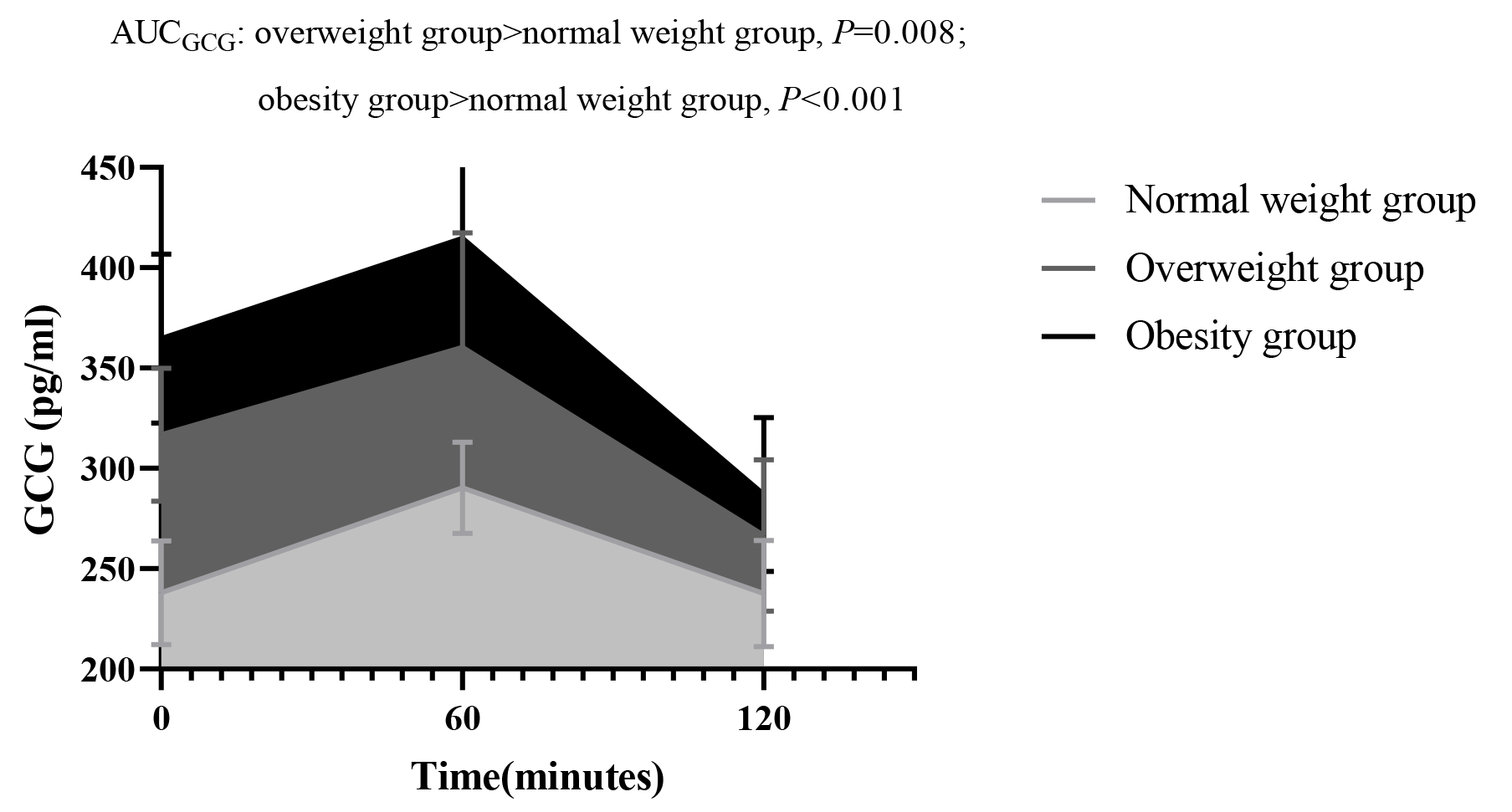

Supplement: Supplementary Figure 2 — The area under curves for the GCG. Multiple comparisons between groups showed that AUCGCG in the obese and overweight group was higher than that in the normal-weight group, and the differences were statistically significant (212.34pg·h/mL, 95% confidence interval 113.94-310.74, p<0.001; 123.72pg·h/mL, 95% confidence interval 32.67-214.76, p=0.008). The difference between obese group and overweight group was not significant (P>0.05). AUCGCG, the area under the curve of glucagon [file Image_2.tif]

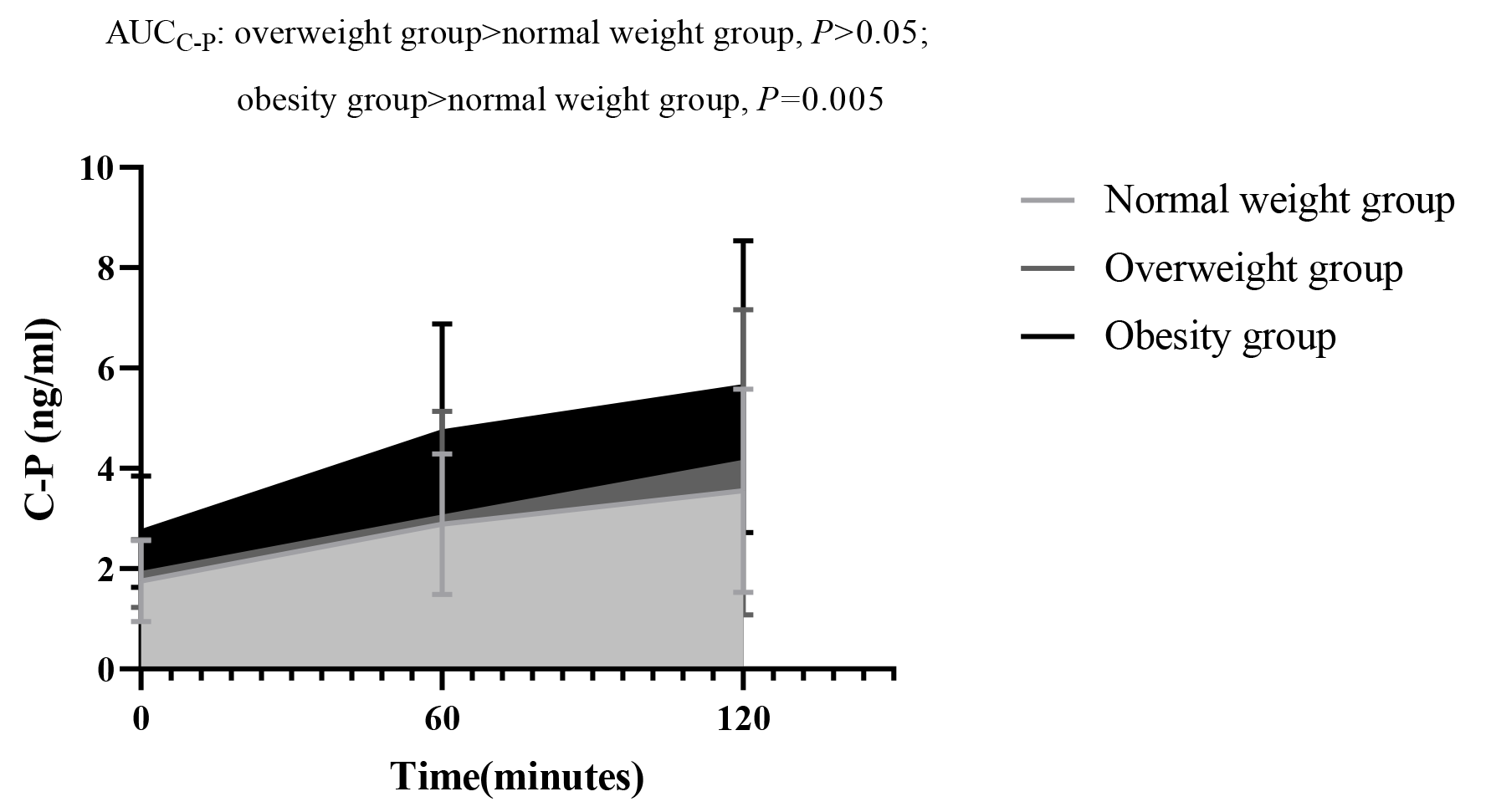

Supplement: Supplementary Figure 3 — The area under curves for the C-P. Multiple comparisons between groups showed that AUCC-P in the obese group was higher than that in the normal-weight group, and the differences were statistically significant (27.62ng·h/mL, 95% confidence interval 8.55-46.69, p=0.005). AUCC-P in the obese group was higher than that in the overweight group, and the differences were statistically significant (25.87ng·h/mL, 95% confidence interval 7.08-44.65, p=0.008). The difference between overweight group and normal-weight group was not significant (P>0.05). AUCC-P, the area under the curve of C-peptide [file Image_3.tif]

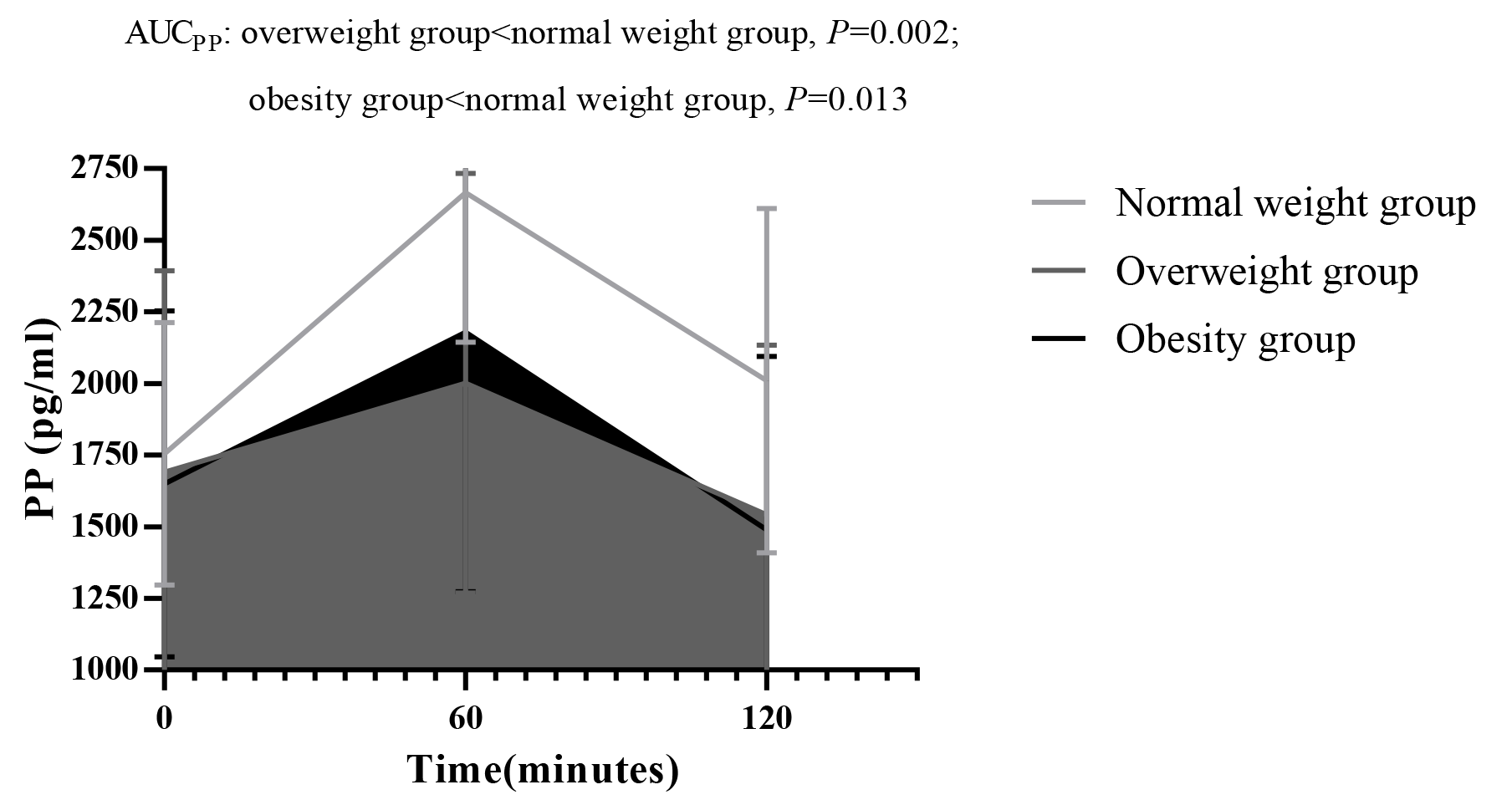

Supplement: Supplementary Figure 4 — The area under curves for the PP. Multiple comparisons between groups showed that AUCpp in the obese and overweight group was lower than that in the normal-weight group, and the differences were statistically significant (159.79pg·h/mL, 95% confidence interval 35.14-284.44, p=0.013; 186.08pg·h/mL, 95% confidence interval 70.75-301.41, p=0.002). The difference between obese group and overweight group was not significant (P>0.05). AUCpp, the area under the curve of pancreatic polypeptide [file Image_4.tif]
